# Human Physiology

**Published:** 1850-10

**Authors:** 


*Human Physiology.* By Robley Dunglison, M. D., Professor of the Institutes of Medicine in Jefferson Medical College, Philadelphia, Vice President of the Sydenham Society, London, &c., &c. *With nearly five hundred illustrations. Seventh Edition. Thoroughly revised, and extensively modified and enlarged.* In two vols. Philadelphia: Lea & Blanchard, 1850.

In our last number we announced a fourth edition of the well-known work on Materia Medica and Therapeutics by the author of the above treatise, and close upon it follows a seventh edition of the "Human Physiology," thoroughly revised and modified—a labor which those only who have kept pace with the rapid strides of Physiology, and the kindred sciences of General Anatomy and

Organic Chemistry, can appreciate. The author informs us that the whole work has been subjected to a rigid scrutiny, both as to the doctrines contained in previous issues, and the language in which they were conveyed, and the result is a work of all the evenness of style so much to be desired in the production of a treatise to be placed in the hands of the younger portion of scientific inquirers. Some idea of the labor expended upon this edition may be had from noting, that the bibliographical list of authors referred to in its preparation extends over nine closely-printed pages, and includes all that has appeared, either in distinct treatises or monographs, since the last edition. As to the manner in which the work is executed, those who are familiar with previous editions need but to be told that this is fully brought up to the day, and presents faithfully the existing state of the science. Where all is excellent it is hard to particularize; we therefore commend the book to the student as the best exponent of the science of Physiology with which we are acquainted, and can heartily sympathize with Dr. Dunglison in the satisfaction with which he has produced a work which redounds so much to his credit, and to that of the profession of which he is so valued a member. The typographical execution is excellent. We notice that many of the old cuts have been replaced by new ones, while additional ones have been introduced, thus greatly enhancing the value of the work.

